# The regulation of intrahepatic fatty acid partitioning within the human liver: the effect of sex

**DOI:** 10.1042/CS20260180

**Published:** 2026-05-22

**Authors:** Kaitlyn M.J.H. Dennis, Coco A. Taylor Start, Dona Josh, Leanne Hodson

**Affiliations:** 1 Oxford Centre for Diabetes, Endocrinology and Metabolism, University of Oxford, Churchill Hospital, Oxford OX3 7LJ, U.K; 2 National Institute for Health Research Oxford Biomedical Research Centre, Oxford University Hospital Trusts, Oxford, U.K

**Keywords:** cardiometabolic disease, fatty acids, Liver, sex, triglycerides

## Abstract

The liver plays a central role in systemic fatty acid (FA) metabolism through the coordinated regulation of hepatic FA uptake, partitioning within, and export. Increasing evidence indicates that hepatic FA metabolism is sexually dimorphic and this may, in part, contribute to sex-specific differences in intrahepatic triglyceride (IHTG) accumulation and susceptibility to metabolic dysfunction-associated steatotic liver disease (MASLD). The sex-dependent divergences in hepatic FA metabolism are thought to arise from differences in systemic FA metabolism, adipose tissue distribution, and intrahepatic FA metabolic pathways, mediated by sexually dimorphic hormonal factors. Here, we review the evidence from human studies and, where appropriate, integrate findings from pre-clinical rodent and *in vitro* cellular models to elucidate how sex influences fatty acid delivery to, synthesis and partitioning with and disposal (through oxidation and secretion as triglyceride in very low-density lipoprotein) from the liver, in a manner that may result in divergent metabolic responses between men and women, potentially leading to dysregulated hepatic metabolism and an altered risk of cardiometabolic disease.

## Overview of human liver fatty acid metabolism

The liver, the largest internal organ in the human body, plays a prominent metabolic role as the gatekeeper of systemic fatty acid (FA) homeostasis. In health, the liver is metabolically flexible, allowing it to rapidly switch between nutrient storage and energy production in response to changes in nutritional and hormonal status [1]. It is also one of the more sexually dimorphic organs in the human body, with FA uptake into, synthesis and partitioning within, and export from the liver appearing to be regulated in a sex-dependent manner, which may, in part, explain divergences between men and women in risk of cardiometabolic disease [2].

In humans, a constant hepatic FA influx is sustained by multiple sources over the course of a day. These include non-esterified fatty acids (NEFA) released during adipose tissue triglyceride (TG) hydrolysis and dietary fats delivered to the liver as chylomicron remnants following peripheral lipolysis and recycled FA from very-low-density lipoprotein (VLDL) particles through uptake of VLDL-TG remnants. Contributions from the NEFA pool to the liver also come from chylomicron-derived FA spillover, which has been postulated to have a larger contribution than VLDL-TG remnants [3,4]. In addition, there is intrahepatocellular synthesis of FAs from non-lipid precursors through the *de novo* lipogenesis (DNL) pathway [1].

Circulating NEFAs bound to albumin enter hepatocytes through regulated transport mechanisms mediated primarily by FA translocase CD36 (FAT/CD36) and the FA transport proteins, FATP2 and FATP5. The liver takes up FAs as TGs through receptor-mediated endocytosis predominantly via members of the low-density lipoprotein (LDL) receptor family, which are hydrolysed by hepatic lipase (HL) and adipose triglyceride lipase (ATGL) [5,6]. Once internalised into the cytosol, FAs are rapidly activated to form acyl-CoA thioesters, which serve as substrates for the synthesis of complex glycerolipids such as TGs and phospholipids [5] or are directed toward mitochondrial β-oxidation.

FAs are exported from hepatocytes as TG through secretion in VLDL. VLDL synthesis occurs in the endoplasmic reticulum, where microsomal TG transfer protein facilitates the transfer of TGs to nascent apolipoprotein B-100 (ApoB100), forming a primordial VLDL_2_ particle. A second lipidation step is required for the maturation of VLDL_1_ particles. Mature VLDL_1_ particles are transported via vesicle-mediated trafficking to the Golgi apparatus before being released into the circulation through specialised transport vesicles [7].

In the liver, the partitioning of FAs among metabolic fates is tightly regulated [8]. Dysregulation in hepatic FA delivery, uptake, synthesis and/or disposal drives enhanced intrahepatic TG (IHTG) accumulation [9,10]. Pathological accumulation of IHTG is an initiating event underpinning the development of metabolic dysfunction-associated steatotic liver disease (MASLD), which is a bidirectional risk factor for type 2 diabetes [11] and an independent risk factor for cardiovascular disease [12]. Therefore, perturbations in hepatic FA metabolism have the potential to have detrimental impacts on whole-body metabolic health.

Hepatic FA metabolism is influenced by genotype and phenotype, as well as physiological and nutritional states, with emerging work suggesting that hepatic FA metabolism may also be strongly influenced by sex; however, conclusive evidence in humans is somewhat limited. Sex-specific differences have been observed in hepatic FA handling, including differences in FA uptake, DNL, mitochondrial β-oxidation, and VLDL secretion [3,13–15] and it has been suggested that sex-dependent differences contribute to sex-based disparities in IHTG content and cardiometabolic risk [16,17]. While genetics (e.g., PNPLA3-I148M variants) strongly influence MASLD risk and IHTG development, sexual dimorphism of the effects of such variants has been reviewed elsewhere [18] and, although of interest, is out of the scope of this review. Therefore, the aim of this review is to integrate findings from human studies and, where appropriate, evidence from pre-clinical rodent and *in vitro* cellular models to elucidate how sex influences FA delivery to, synthesis and partitioning with and export from the liver, in a manner that may result in divergent metabolic responses between men and women, potentially leading to dysregulated hepatic metabolism and an altered risk of cardiometabolic disease.

## Sex differences in MASLD risk and prevalence

MASLD is the most common chronic liver condition characterised by excessive IHTG accumulation in the presence of one or more cardiometabolic risk factors [19] and recent work has indicated that sex plays a major role in MASLD development through influencing IHTG dynamics.

Globally, MASLD prevalence shows significant sex- and age-specific patterns. Men have a higher prevalence of MASLD compared with age-matched premenopausal women [20]. Prospective cohort studies support greater MASLD incidence rates in men compared with women [21–23], indicative of greater MASLD risk in men.

There is also evidence supporting sexual dimorphism in the risks of some of the cardiometabolic traits associated with MASLD, which may be influenced by the transition to menopause in women. Overall women appear to have a lower risk of developing type 2 diabetes than men [24]; however, the menopausal transition is associated with an increased incidence rate in women [25,26]. Conversely, prospective cohort studies find that women have a greater incidence rate of hypertension [27].

In men, MASLD prevalence increases during adulthood, peaking between 50 and 60 years before a subsequent decline. In comparison, premenopausal women have a lower prevalence of MASLD, which then increases after the age of 50, with postmenopausal women having similar or higher rates of MASLD compared with age-matched men [28]. Although the prevalence data would suggest a role for oestrogen in protecting against MASLD development, data for menopause-specific MASLD are lacking, and the mechanisms governing any potential differences are not yet fully understood. The increased prevalence of MASLD in women post-menopause may, in part, be explained by the changes in whole-body fat distribution with a shift in fat storage from lower-body subcutaneous stores to upper-body or visceral fat depots [29,30]; the latter being significantly associated with an increased risk of myocardial infarction [31]. Moreover, work from preclinical models has suggested a role for oestrogen in protecting against insulin resistance [32] and oxidative stress [33,34], both of which have been suggested to play a role in MASLD development [35,36].

As IHTG accumulation underpins the development of MASLD, understanding the sex-dependent mechanisms regulating IHTG levels and hepatic FA metabolism is important for the development of future therapies.

## Sex differences in hepatic FA partitioning

There has been very little work investigating sex differences in hepatic FA influx or partitioning in humans, likely due to the challenges associated with complex *in vivo* human physiological studies which indirectly assess intrahepatic FA partitioning. Furthermore, human metabolism is dynamic and may be altered by many factors, including phenotype, age, diet, and exercise. Although tracking changes in hepatic FA metabolism within a person across a lifespan would be highly informative, it is experimentally difficult. Thus, the majority of available evidence comes from observational studies.

## Hepatic FA sources: adipose tissue-derived NEFA

Plasma NEFAs are a key source of FAs for the liver, contributing to the majority of VLDL-TG production by the liver during fasting [37]. Some [38–43], but not all [15,44,45], studies have reported fasting NEFA concentrations to be higher in women compared with men. In the transition to the postprandial state, after consumption of an experimental test meal, some suggest no difference [4,15] in systemic NEFA concentrations; however, others have reported men to have higher postprandial NEFA concentrations than women [46,47]. This discrepancy may in part be explained by a higher NEFA rate of appearance in men when compared with pre-menopausal women [48], suggesting differences in insulin-mediated suppression of AT lipolysis between sexes. The discrepancy in findings is potentially due to the high variability observed in fasting plasma NEFA concentrations within individual participants [49,50] amplified by the relatively small sample sizes of many *in vivo* human studies. Differences between studies in the period of fasting prior to measuring plasma NEFA concentrations, phenotype of participants, and test meals given all potentially contribute to the discrepancies observed between studies. Stable-isotope tracer studies in humans have found plasma NEFA to be a key contributor to liver FA flux in the postprandial state [51–53], with approximately 60% of liver TG resulting from adipose tissue-derived NEFA in people with MASLD [54]. There are, however, clear differences in postprandial NEFA kinetics, with women displaying a greater rate of appearance and disappearance than men [46].

Adipose tissue metabolism plays a key role in regulating systemic FA availability, which has consequences for hepatic FA metabolism. In the postprandial state, adipose tissue takes up and stores dietary FAs largely through the hydrolysis of circulating chylomicron and VLDL-TG via the action of lipoprotein lipase, with FAs that escape uptake spilling over into systemic circulation. During fasting, adipose tissue-derived NEFAs account for the majority of FAs in VLDL-TG released from the liver [37] (Table 1).

**Table 1:** Fasting plasma NEFA concentrations in women and men

Study	Subjects	Age (years)	BMI (kg/m^2^)	NEFA concentrations
Bakewell et al. [38]	23W	23 ± 4^a^	22.7 ± 2.3^a^	209 ± 92^†a^
	13M	26 ± 5^a^	23.3 ± 2.8^a^	79 ± 48^†a**^
Marinou et al. [40]	12W	22.5 ± 2.2^b^	21.0 ± 1.6^b^	557 ± 88^‡b^
	12M	25.5 ± 4.4^b^	21.5 ± 2.5^b^	296 ± 26^‡b*^
Frayn et al. [41]	87W	NR	18.3–53.4^c^	655 ± 20^‡b^
	166M	NR	18.3–53.4^c^	532 ± 14^‡b**^
Pramfalk et al. [15]	11W	46 ± 6^b^	28 ± 3^b^	488 ± 121^‡b^
	11M	46 ± 7^b^	27 ± 2^b^	451 ± 108^‡b^
Magkos et al. [44]	13W	28 ± 6^a^	22.0 ± 15^a^	574 ± 271^‡a^
	13M	29 ± 5^a^	22.0 ± 1.7^a^	397 ± 154^‡a^
Soeters et al. [42]	10W	22.2 (19.1–28.9)^d^	22.9 (18.7–24.2)^d^	1.26 (0.93–1.54)^¥d^
	10M	21.3 (18.9–25.1)^d^	21.5 (19.2–24.7)^d^	0.96 (0.72–1.18)^¥d*^
Shitole et al. [45]	1294W	77.7 ± 4.4^e^	26.8 ± 4.8^e^	0.4 ± 0.2^¥e^
	850M	78.1 ± 4.6^e^	26.5 ± 3.7^e^	0.3 ± 0.1^¥e^
Chrzanowski-Smith et al. [43]	49W	33 (44)^d^	22.5 ± 2.4^a^	0.42 (0.90)^¥d^
	50M	37 (39)^d^	24.7 ± 2.8^a^	0.31 (0.87)^¥d*^

Abbreviations: W, women; M, men; BMI, Body Mass Index; NEFA, non-esterified fatty acids; NR, not reported; ^a^data presented as mean ± SD; ^b^data presented as mean ± SEM; ^c^data presented as minimum–maximum; ^d^data presented as median (range); ^e^data presented as median ± SD; NEFA concentrations as ^†^μg/ml; ^‡^μmol/L; ^¥^mmol/L. ^*^P <0.05; ^**^P <0.001 between women and men within the same study.

Body composition, specifically proportions of lean and fat mass, plays a pivotal role in regulating NEFA flux. In humans, at a given body mass index (BMI), men typically have more fat-free and less adipose tissue mass and therefore a higher total body energy expenditure, even after adjusting for fat-free mass, compared with women [55,56]; these differences may underpin the divergences in hepatic FA influx. Mittendorfer et al. [57] found the rate of NEFA release into plasma per unit of fat mass was similar in men and women but the total rate of release in relation to fat-free mass was greater in women than men, as women have more body fat than men. NEFA release per unit of fat mass has been found to decrease with increasing adiposity in both men and women [50,57].

We have previously observed greater chylomicron-derived spillover in women compared with age- and BMI-matched men, which does not appear to be the result of the increased adiposity, as we have previously observed significantly lower chylomicron-derived spillover in obese compared with lean individuals [4]. We postulated that lower chylomicron-derived spillover is due to reduced adipose tissue blood flow leading to a delayed clearance of chylomicrons, which allowed for greater uptake of the FA released by lipoprotein lipase [4,58]. The higher chylomicron-spillover-derived FA observed in women is suggested to be the result of faster chylomicron hydrolysis, which results in a reduced ability for the entrapment of NEFA released during chylomicron lipolysis in premenopausal women [59].

Differences in total adiposity (women typically having approximately 10% more body fat compared with men [60]) and in adipose tissue distribution may contribute to sex differences in NEFA kinetics. Women have been reported to have more abdominal and gluteofemoral subcutaneous adipose tissue (SAT) and lower amounts of visceral adipose tissue (VAT) than men with similar levels of total adiposity [60]. Using arterio-venous difference techniques across abdominal SAT, Frayn and Humphreys [41] found no difference in fasting NEFA release (nmol·100 g^−1^·min^−1^) or the percentage re-esterification between men and women, although they noted women had significantly higher fasting TG fractional extraction than men (7.1 ± 0.8% compared with 4.9 ± 0.4% (mean ± SEM), respectively). *In vivo* human postprandial studies using isotope tracer methodologies found that premenopausal women store a greater proportion of dietary FAs in gluteofemoral SAT, while men primarily store dietary FAs in VAT [61–63]. Furthermore, it has been found that insulin-mediated suppression of adipose tissue lipolysis, following meal consumption, occurs to a greater extent in women compared with men [48], suggesting women have a reduced flux of adipose tissue-derived FAs to the liver following meal consumption.

Adipose tissue depots differ in lipolytic rate and this may influence IHTG accumulation in humans [64]. Work in humans and *in vitro* cell models has demonstrated that the rate of lipolysis is lower in the gluteofemoral SAT compared with abdominal SAT and VAT [65] as well as being more readily suppressed by insulin [66]. Moreover, VAT-derived NEFAs go directly to the liver via the portal circulation, while FAs released from SAT enter the liver via systemic veins [67]; it has been suggested that men may have a higher flux of NEFA to the liver, due to typically having a higher amount of VAT, than women. However, the contribution of NEFA from VAT to total NEFA uptake by the liver is suggested to be relatively limited [37] and is unlikely to be a considerable driver of sex differences in NEFA flux to the liver [67].

While there are higher relative rates of NEFA release in women, these appear to be matched by higher clearance rates in the form of NEFA oxidation (oxidative clearance) and NEFA uptake and storage (non-oxidative clearance) at the whole-body level [46]. Romanski and colleagues [62] measured the uptake of meal FAs into abdominal, gluteal, and thigh SAT in young, healthy, lean men and age- and BMI-matched premenopausal women, 24 h after meal ingestion. They found a similar uptake into abdominal and gluteal with a lower uptake into thigh SAT in both men and women. However, when uptake was corrected for regional fat mass, men had a significantly lower uptake into upper- and lower-body SAT, with a significantly lower proportion being stored in total SAT, than premenopausal women. As there were no sex differences in the oxidation of the meal FAs, it was suggested that the unaccounted meal FAs were either stored in VAT or as ectopic TG [62].

Adipose tissue depots have different capacities for adipose tissue expansion. White and colleagues [68] utilised stable-isotope-labelled water to study the *in vivo* cellular kinetics of abdominal and femoral SAT in women with overweight or obesity. They found that femoral SAT had a higher capacity for adipogenesis compared with abdominal SAT, suggesting women may have greater adipogenic capacity than males, due to their propensity to have more femoral SAT. Even within the same AT depot, sex differences may exist in adipose tissue expansion. Tchoukalova et al. [69] noted that, when healthy (average BMI 22.1 kg/m^2^) men (*n* = 15) and women (*n* = 13) gained approximately 3.8 kg of fat tissue by consuming excess calories for 8 weeks, men had a greater increase in upper body SAT, which was suggested to be due to hypertrophy, while women had a greater increase in lower body SAT, which was suggested to occur through hyperplasia, compared with men. The potential bias in adipogenic capacity in lower body SAT in women may result in a lower NEFA flux to the liver, even when there is an increase in total body fat mass. Hypertrophied adipose tissue has been shown to have reduced capacity for fat storage, resulting in enhanced NEFA spillover [70], which may be directed to the liver, potentially driving enhanced IHTG accumulation. Taken together, sex-specific patterns of fat distribution may influence hepatic FA metabolism through regulation of FA flux from adipose tissue to the liver.

## Hepatic FA sources: triglyceride-rich lipoproteins and spillover FAs

TG-rich lipoproteins (TRLs) broadly encompass intestinally derived chylomicrons, liver-derived VLDLs and their partially hydrolysed ‘remnants’. They provide an alternative lipoprotein-mediated route of FA entry into the liver, particularly in the postprandial state when they have the potential to carry a high proportion of dietary FAs. For example, for individuals with dysfunctional adipose tissue, like those with insulin resistance, TRLs may not be as well lipolysed by adipose tissue lipoprotein lipase compared with insulin-sensitive individuals with functional adipose tissue, and this leads to a potentially increased contribution of TG to the liver due to ineffective storage of dietary fats in adipose tissue [58].

Some variation in the exact definitions of ‘remnants’ [71–75] and TRLs [47,71] exists in the literature, adding difficulty to comparison between studies. Experimentally, the cut-off of Svedberg flotation (S_f_) >20 is often used to define TRLs, with the particles with S_f_ >400 being considered chylomicrons and those between S_f_ 20 and 400 considered chylomicron remnants and VLDL [47,76,77], although this likely does not encompass many small VLDL remnants [78]. However, the relevance of excluding smaller VLDL remnants for studies of hepatic FA metabolism is unclear, as they are more TG depleted and, as such, will likely contribute relatively little FA to the liver. The sparsity of research into the implications of TRLs on liver fat metabolism, likely in part due to a lack of clarity in their definition, is amplified when considering sex differences in TRL metabolism.

**Figure 1:**
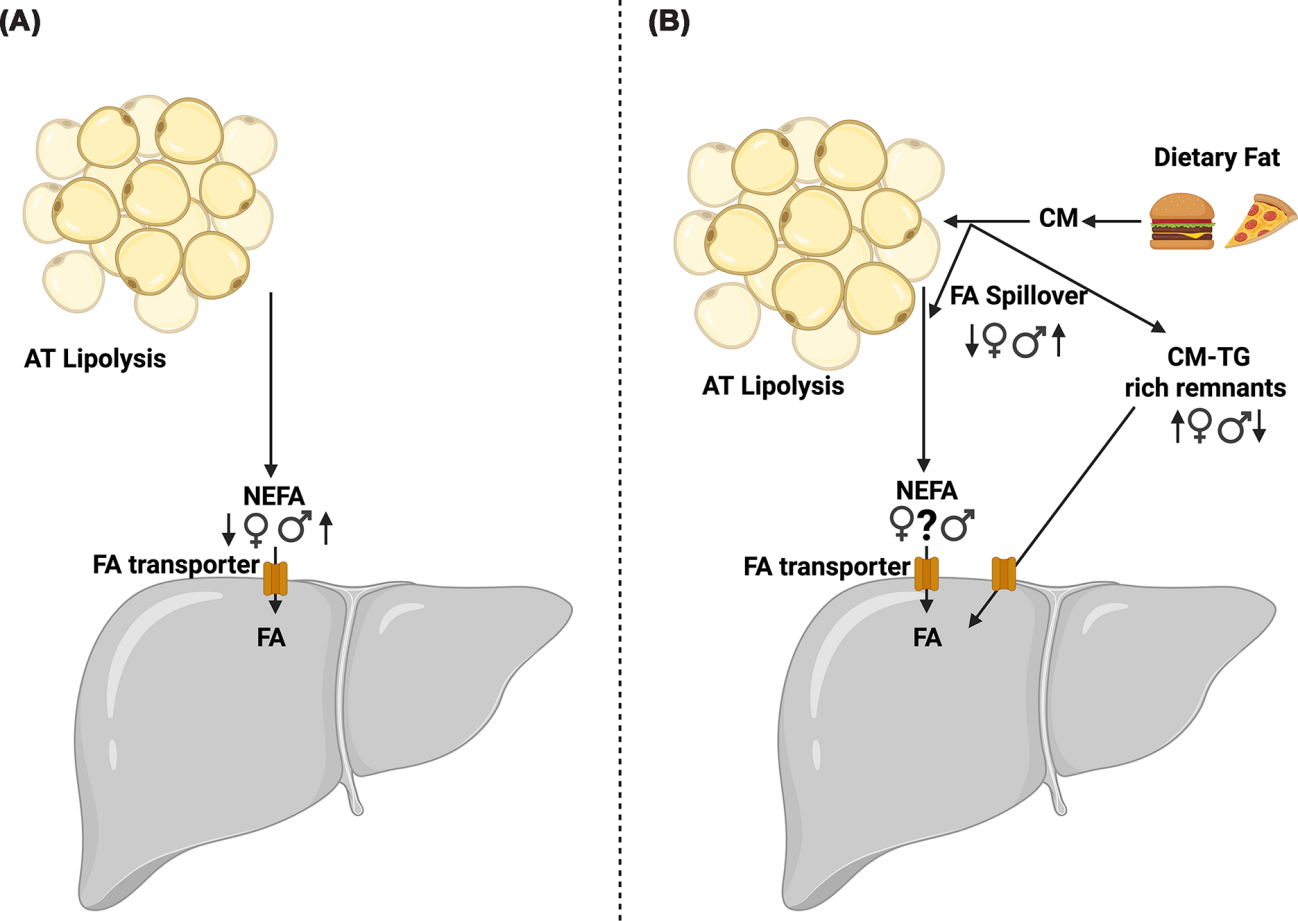
Overview of sex differences in fatty acid and triglyceride trafficking to the liver in the fasting and postprandial state. In the (**A**) fasting state, women tend to have higher systemic NEFA concentrations than men, which may lead to a greater FA flux to the liver. In the transition to the (**B**) postprandial state, although it remains unclear if men and women, when fed an experimental test meal, achieve similar systemic NEFA concentrations, chylomicron-derived fatty acid spillover has been reported to be higher in women compared with men. Figure created in https://BioRender.com. Dennis, K. (2026) https://BioRender.com/0gws00e. Symbols and abbreviations: ♀ premenopausal women; ♂ men; ?, unclear due to divergent findings; =, no difference between sexes; AT, adipose tissue; NEFA, non‐esterified fatty acid; TG, triglyceride; CM, chylomicrons; FA, fatty acid.

While research is limited, sex differences in adiposity and plasma NEFA concentrations suggest sexual dimorphism in TRL-TG concentrations and different partitioning of TGs with the TRL classes. With increased adiposity, higher rates of chylomicron and VLDL lipolysis might be expected, resulting in greater NEFA spillover and more lipolysed TRL remnants (Figure 1). The only study on sexual dimorphism of TRLs (S_f_ > 20) that we can find suggests men have a higher postprandial TRL-TG pool [47] and therefore likely higher TRL-FA delivery to the liver than premenopausal women.

Few studies measure native chylomicrons from the lymph, and there are challenges when measuring systemic chylomicrons, as when determining TG load of the chylomicrons, it is unclear how they are directly produced by the intestine or a result of hydrolysis of the chylomicrons by peripheral tissues. Moreover, feeding men and women the same meal may be proportionately over- or under-feeding one group due to a relative energy requirement mismatch. While the effects have not been directly studied on FA metabolism, when a test meal is scaled to energy expenditure, sex differences in postprandial glucose metabolism between men and premenopausal women are lost [79]. As such, overfeeding women may result in exaggerated chylomicron-TG responses, driving increased chylomicron spillover and remnant formation compared with the same meal fed to men (Figure 1).

VLDLs show greater hydrolysis into intermediate-density lipoproteins (IDLs: partially lipolysed VLDLs [71]) in women than men [46,80], with greater IDL-TG concentrations seen in women [81]. There is evidence suggesting sexual dimorphism in both total TRLs and partitioning of TGs within the TRL classes which may alter rate of FA uptake into the liver; for example, chylomicrons have a larger surface area than VLDLs and, therefore, bind more apolipoprotein E, resulting in greater hepatic uptake [73].

## Hepatic FA transporters and FA uptake

**Figure 2:**
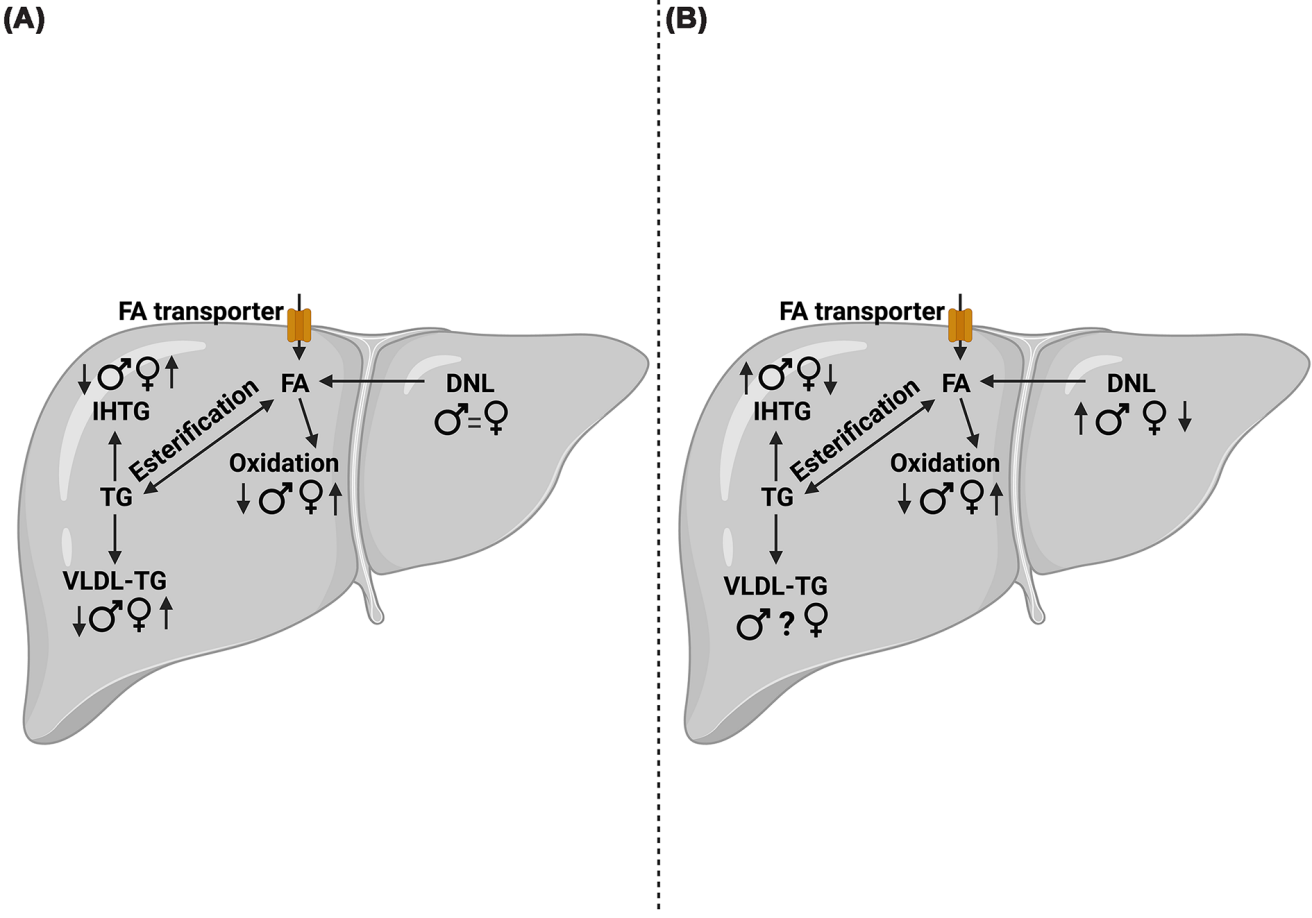
Overview of sex differences in intrahepatic fatty acid partitioning in the fasting and postprandial state. In fasting (**A**), from very limited human data, it is suggested there is no difference in intrahepatic DNL, and markers of fatty acid oxidation and ketogenesis are higher in women, along with higher rates of VLDL-TG secretion and clearance, when compared with men. Postprandially (**B**) hepatic DNL is suggested to be up-regulated to a greater extent in men compared with women, while markers of dietary fatty acid oxidation and ketogenesis are high in women compared with men. It remains to be elucidated where differences exist between men and women in VLDL-TG secretion and clearance in the postprandial state. Taken together these differences may, in part, explain the higher propensity of MASLD in men compared with premenopausal women. Figure created in https://BioRender.com. Dennis, K. (2026) https://BioRender.com/lokrus1. Symbols and abbreviations: ♀ premenopausal women; ♂ men; ?, unclear due to divergent findings; =, no difference between sexes; fatty acid, FA; DNL, *de novo* lipogenesis; VLDL, very-low density lipoprotein; IHTG, intrahepatic triglyceride accumulation.

Much of our understanding of sex differences in hepatic FA uptake comes from rodent studies, where there is clear sexual dimorphism in hepatic FA transporter expression and some functional data (Figure 2). For example, increased FA uptake rate is observed in female compared with male rat livers, with no difference in the surface expression of FABP_pm_ and, therefore, likely an increased functional affinity [82]. Moreover, there is an increased content of cytosolic FABP in female rodent liver, which may increase the capacity for intracellular FA transport [82]. Higher *CD36* mRNA expression is seen in female compared with male rodent livers, accompanied by increased protein expression but no dimorphism in intracellular TG content [83], perhaps a result of more rapid FA efflux and FA oxidation (FAO) from the female liver. A kinetic study noted that rodent female livers have a greater LDL receptor density, with a higher B_max_ compared with males [84]. Taken together, the findings to date suggest enhanced FA uptake in the female rodent liver. How this translates to human metabolism remains unclear, with most human studies being limited to transcriptional data, which supports the rodent data, although it may not be reflected at the protein level or in activity.

There is evidence of higher *CD36* [83] and *LDLR* [85] mRNA expression in female human and rodent livers, which may support a greater capacity for FA uptake in females (Figure 2). In a novel human functional study, dynamic, whole-body positron emission tomography/computed tomography (PET/CT) was used to directly measure FA uptake into liver, with no sex effect on postprandial hepatic NEFA uptake being observed [86]. However, as the present study is limited to post-menopausal women, findings may differ if men and pre-menopausal women were compared. Despite this, in humans, men typically have higher IHTG content than age-matched women [87], suggesting if women have a higher hepatic uptake of FAs, they must be secreting or oxidising them instead of storing them.

## Intrahepatic FA synthesis: *de novo* lipogenesis

DNL provides another source of FA within the liver. From limited data, hepatic DNL does not appear to show sexual dimorphism in the fasted state [15], although in the postprandial period men display a higher contribution of DNL-derived FA to VLDL-TG than women [15] (Figure 2). This observation is indicative of a greater rate of DNL, which may contribute to a greater IHTG accumulation compared with women; however, findings need to be replicated.

Up-regulating DNL leads to an elevation in intrahepatocellular cytosolic malonyl-CoA levels, which may shift metabolism away from oxidation towards esterification [88]. Although it is yet to be demonstrated, it is plausible that the regulation of the malonyl-CoA switch and mitochondrial sensitivity to malonyl-CoA may be sexually dimorphic [14,15,89].

## Hepatic FA disposal: oxidation

Findings for sex differences in whole-body FAO are inconsistent across studies, which may be explained by differences in participant nutritional state and methodology used to assess FAO. Using indirect calorimetry, some have reported lower fasting FAO rates in healthy women compared with men [90,91], whereas others find negligible or no differences [92]. We have used stable-isotope tracers to assess whole-body FAO in the postprandial state and found women to have higher FAO compared with age- and BMI-matched men [15]. Any observed differences in whole-body FAO may be driven by hepatic FAO (Figure 2).

Oxidation of FAs within the liver may be via complete β-oxidation (the tricarboxylic acid (TCA) cycle) and ketogenesis [8], and for the latter, 3-hydroxybutyrate (3-OHB) is the predominant ketone body produced, being almost entirely liver-derived [93], and circulating plasma levels are often used as a surrogate marker of hepatic FAO. Using stable-isotope tracer methodologies, we have previously observed that partitioning of FAs towards 3-OHB in the fasting state occurs to a greater extent in premenopausal, healthy, lean women compared with age- and BMI-matched men [40]. Moreover, we have observed that in the transition to the postprandial state, a greater appearance of ^13^C label, from dietary fat, is incorporated in plasma 3-OHB in women compared with age- and BMI-matched men [15]. Currently, complete hepatic FAO is not measured by many who undertake human studies, although it is suggested that complete hepatic FAO is, at least in rodents, a minimal and relatively constant pathway [8]. Using dynamic PET/CT to assess postprandial hepatic NEFA oxidation, Ye et al. [86] found no difference between men and age- and BMI-matched postmenopausal women. However, oxidative, not just ketogenic pathways, were measured at only one postprandial timepoint, 90 min after the meal, limiting comparison to other work. Thus, findings from the limited data in humans suggest that women, when compared with men, may have higher rates of hepatic FAO, at least in the ketogenic pathway, which could contribute to the lower levels of IHTG accumulation.

## Hepatic triglyceride disposal: very low-density lipoprotein triglyceride secretion and clearance

Within the liver FAs can be partitioned into esterification pathways, where synthesised TG can be stored within or exported from the liver in VLDL (Figure 2). Using PET/CT methodologies, it was found men had higher total and hepatic NEFA esterification than postmenopausal women, which, due to the challenges of measuring directly, was inferred from changes in oxidation and VLDL-TG secretion [86]. It is consistently reported in humans that men have higher fasting plasma VLDL-TG concentrations than premenopausal women, at any degree of adiposity [15,81,94], and although women have a higher VLDL-TG secretion rate, this is balanced by an increased clearance rate and shorter mean residence time [44,83]. The higher VLDL-TG secretion rate in women may contribute to a reduced propensity to IHTG accumulation compared with men. Moreover, women have lower plasma VLDL-ApoB100 concentrations, indicating lower VLDL particle number, resulting from a decreased secretion rate and shorter residence time in the plasma with no difference in clearance rate [44,95].

The VLDL-TG to VLDL-ApoB100 ratio indicates how TG-rich particles are. Women have a notably higher VLDL-TG to VLDL-ApoB100 secretion ratio, indicative of fewer, but more TG-rich, VLDL particles [15,44]. Furthermore, in women with larger, TG-richer VLDL particles, rapid delipidation is observed, reflecting faster clearance rates and short mean residence time [94,96]. Overall, this may help explain the lower proportion of circulating large VLDL to total VLDL particles in women compared with men when blood lipoprotein profiles are measured by proton nuclear magnetic resonance spectroscopy rather than kinetic studies [81,95]. By studying pre- and postmenopausal women, it is suggested that oestrogen deficiency may drive the sexual dimorphism in VLDL-TG secretion but not regulate VLDL-ApoB100 secretion [96].

An alternative to secretion in VLDL for newly synthesised TG is storage in lipid droplets. Modelling sex differences *in vitro* is challenging, and limited to primary cells and induced pluripotent stem cells, as current cell lines (HepG2 and Huh7) are male-derived. *In vitro* experiments are typically performed with a small sample size, and primary human hepatocytes (PHHs) are dependent on donor and disease state; as such, applicability of these small-scale *in vitro* studies to human *in vivo* work is somewhat unclear. There has been increasing focus on the need to use both male and female preclinical rodent models; however, the same focus has not yet been given to *in vitro* models. Many studies do not report the sex of primary cells and cell lines, and even fewer use a mixture of both male and female cells [97]. A key benefit of cell culture models in the study of sex differences is the ability to separate the impact of cell sex from different sex hormones and the ability to study the influence of the sex hormones themselves. *In vitro* studies suggest no difference between IHTG content between male and female PHHs, with an albeit increased apoB100 (VLDL) secretion rate in female PHHs when compared with males [83]. Seidemann and colleagues [83] found that NEFA exposure, to induce IHTG accumulation, results in sexually dimorphic effects on lipid droplet morphology. In female PHHs, increased IHTG was the result of a greater accumulation of small lipid droplets, whereas in male PHHs, a greater proportion of large lipid droplets was seen [83], although there appears to be greater variation in female PHHs, which may be due to the small sample size (*n* = 2 or 3) or perhaps reflective of a greater ability to alter FA flux through the liver to reduce excess FA accumulation. The observations could also reflect donor life stage, as the age range for female PPHs was 46 to 65 years old, likely reflecting a mixture of pre- and postmenopausal female donors [83]. The discrepancies between the *in vitro* and *in vivo* human work highlight the relevance of systemic metabolism in influencing hepatic VLDL-TG dynamics.

*In vivo* human studies show in premenopausal women intrahepatic FA partitioning appears to favour FAO and an increased VLDL-TG secretion rate, with the secretion of more TG-rich VLDL, while in men there appears to be a preference for partitioning of FAs towards esterification and storage of TG, perhaps a result of a mismatch between the rates of FA import into the liver and export and/or oxidation. This imbalance may, in part, drive an increased propensity for IHTG accumulation in men compared with premenopausal women.

## Sexual dimorphism in hormonal regulation of liver metabolism

The pivotal role of the liver in maintaining systemic metabolic FA homeostasis is strongly influenced by the action of key hormones, including insulin, glucagon, and the sex hormones oestrogen and testosterone. Circulating concentrations of these hormones can differ substantially between men and women, and fluctuations in levels elicit distinct, sex-dependent metabolic responses that modulate the balance among FA influx, intrahepatic partitioning, and efflux from the liver.

Insulin and glucagon, produced by pancreatic β- and α-cells, respectively, control liver substrate flux by regulating the switch between the catabolic and anabolic states following their rapid delivery to the liver by the hepatic portal vein [98]. They are key regulators of hepatic FA flux [8] with insulin inhibiting adipose tissue lipolysis [37], reducing FA flux to the liver in the postprandial state. This is accompanied by a metabolic shift in hepatic FA metabolism towards DNL and esterification, and away from FAO by increasing malonyl-CoA, leading to the inhibition of the carnitine shuttle.

Sex differences have been noted in pancreatic morphology, with women typically having a smaller pancreas than men [99] with either no difference [100] or a modest increase in β-cell content in women compared with men [101]. It is plausible the inconsistent findings are due to differences in the techniques used to assess β-cell content and/or being underpowered to measure modest differences within the pancreas; there is regional heterogeneity in β-cell content with additional individual variability [102], further complicating measurements.

Functional studies in humans and rodents support a greater postprandial insulin secretion in females than in males [100,103,104]. *In vitro* studies suggest greater exocytosis of insulin granules from female islets, with more membrane-docked granules [104] and increased electrical activity due to smaller Kv2.1 channels [105], which has been postulated to arise through strong sexually dimorphic epigenetic divergences in insulin secretion [100]. In addition to insulin secretion, premenopausal women are suggested to have better whole-body insulin sensitivity compared with men [106,107]. Overall, it appears that women show greater insulin secretion and sensitivity.

Evidence surrounding differences in glucagon secretion in men and women is unclear. Some human studies have reported an absence of significant sex effects, albeit with trends acknowledged [104,108], while others suggest that women appear to show reduced glucagon suppression following glucose challenge [103] and reduced glucose secretion in response to hypoglycaemia [109] compared with men. It is, however, thought sex differences in catecholamines may play a more significant role in fasting blood glucose concentrations than glucagon [110].

Age-related alterations to pancreatic hormone secretion are also sexually dimorphic. Female islet cells show reduced insulin and glucagon secretion with age, while male islet cells increase insulin secretion with no change in glucagon secretion [104] and are accompanied by physiological evidence supporting sexually dimorphic, altered postprandial glucose concentrations with age [111].

The increased insulin secretion in women is interesting when considering hepatic FA partitioning, as it might be expected increased insulin secretion and potentially suppressed glucagon response seen in women would result in greater FA esterification and storage and reduced FAO, driven by an up-regulation in malonyl-CoA, when compared with men. It is plausible these observations may partly be explained by sexual dimorphism in the malonyl-CoA switch [14,15,89]. It is also possible that DNL substrate limitation may occur in women. For example, DNL could be initially up-regulated in women in response to the greater insulin secretion and sensitivity; however, if there is reduced hepatic substrate content in women, including glucose, fructose and amino acids, DNL could be down-regulated, leading to a subsequent up-regulation of FAO. Work on sexual dimorphism in hepatic substrate availability for DNL is limited, with sex differences in hepatic glycogen, for example, currently restricted to rodent studies [112], therefore adding difficulty to elucidating the mechanism behind this perhaps unanticipated hepatic DNL response in women.

## Effect of sex hormones on liver FA metabolism

### Oestrogen

Within the liver, oestrogen signalling is a tightly coordinated process, influenced by circulating and locally synthesised oestrogen levels, along with the expression of hepatic oestrogen receptors [113]. In humans, women produce four major endogenous oestrogens: oestrone (E1), 17β-oestradiol (E2), oestriol (E3), and oestriol (E4), while men produce E1 and E2. In premenopausal women, E2 is synthesised from cholesterol in the ovaries, while in men and postmenopausal women, E2 is synthesised from the aromatisation of testosterone in peripheral tissues [114]. Work in humans has found E1 and E2 levels in women increase with age and pubertal stage, and fluctuate during the menstrual cycle [115]. E2 is the predominant and most potent oestrogen in women before the menopause, whereas E1 shifts as primary endogenous oestrogen in postmenopausal women [115]; however, the mechanisms and importance remain unclear. In men, E1 and E2 also rise with age and pubertal stage, but remain relatively stable thereafter [115].

Evidence in humans implicates oestrogen in regulating whole-body and hepatic FA metabolism. In a longitudinal study in premenopausal women, changes in body fat distribution and FAO (using 24-h whole room calorimetry) were assessed over time. It was found that women who became postmenopausal increased VAT mass and decreased whole-body FAO compared with their premenopausal measurements [30]. In contrast, women who did not undergo menopause over the course of the study had an increase in adiposity, but it was attributed to increases in SAT depots, and FAO levels remained unchanged [30], indicating that oestrogen may play a role in regulating whole-body FAO and body composition. In further support of oestrogen regulating FAO, men undertaking endurance exercise, who were supplemented with E2 had an increase in whole-body FAO and decreased carbohydrate oxidation [116]. However, not all reports in humans demonstrate that oestrogen influences whole-body FAO. Findings from pre- and postmenopausal women matched for abdominal obesity and liver fat found no differences in fasting whole-body FAO rates as measured by stable-isotope tracer methodology [117]. Divergences in findings between the studies may be explained by differences in the study cohort (e.g., age and phenotype) and nutritional state (fed versus fasted).

*In vitro* cellular work found the application of oestrogen to female PHHs decreased intrahepatocellular TG levels by reducing lipid droplet size [83]. Premenopausal women with clinically diagnosed low oestrogen levels and those undergoing oophorectomies are reported to have a higher prevalence of MASLD compared with premenopausal women with clinically normal oestrogen levels [118,119]. It is becoming evident that in postmenopausal women, where E2 levels are reduced, there is an increased risk of IHTG accumulation and development of MASLD that is comparable to age- and BMI-matched men [120]. Additionally, it is suggested that premenopausal women with hormone-sensitive breast cancer who take the anti-oestrogen drug tamoxifen develop liver steatosis [121,122]. In contrast, a very limited number of studies have suggested that increased oestrogen levels in men may be detrimental to risk of MASLD [123,124]. Taken together, it appears that oestrogen may directly influence intrahepatocellular TG content by influencing lipid droplet dynamics.

The mechanisms of oestrogen signalling in regulating whole-body and hepatic FA metabolism have been extensively explored in female rodent models. Findings from surgically ovariectomised (OVX) rodents, which consistently display reduced whole-body FAO [125,126], are also accompanied by increased insulin resistance, IHTG accumulation, and dyslipidaemia [127–132]. The increase in hepatic IHTG content observed in OVX mice has been proposed to result from impaired hepatic VLDL-TG efflux, as reductions in both VLDL-TG production and microsomal transport proteins have been reported [133]. Furthermore, in rodents, E2 replacement at the time of ovariectomy reduces hepatic IHTG accumulation, an effect attributed to decreased DNL and preserved VLDL-TG efflux [125,127,134,135]. Based on these findings, oestrogen appears to be a regulator of hepatic FA metabolism, potentially through regulating VLDL-TG synthesis and secretion, and it could be speculated from the findings from E2 treatment in OVX rodent models that E2 could be useful as HRT in humans (Table 2). Oestrogen signalling is mediated by the nuclear hormone receptors oestrogen receptor (ER) alpha (ERα) and ER beta (ERβ), and the membrane-bound receptor G-protein coupled oestrogen receptor 1 (GPER1) [136]. In the liver, ERα and GPER1 are the primary oestrogen receptors in humans and rodents of both sexes, while ERβ is minimally expressed. In rodents, it has been shown that ER expression is not sex-dependent, but age-dependent, with highest levels in the perinatal and postpubescent periods [137,138]; however, this has yet to be explored in humans.

**Table 2:** Proposed effects of oestrogen and testosterone levels on hepatic FA handling in women and men and preclinical female and male rodent models

♀	Low Oestrogen	Physiological Oestrogen	Physiological testosterone	High Testosterone
IHTG	↑	↓	?	↑
VLDL-TG Efflux	↓	↑	?	?
FAO	↓	?	?	?
DNL	↑	?	?	?

♂	Low Oestrogen	Physiological Oestrogen	Low Testosterone	Physiological Testosterone
IHTG	↑	↓	↑	↓
VLDL-TG Efflux	?	?	↓	?
FAO	?	?	↓	?
DNL	↑	?	↑	?

Abbreviations: ♀, women/female; ♂, men/male; IHTG, intrahepatic triglyceride accumulation; VLDL-TG, very low-density lipoprotein–triglyceride; FAO, fatty acid oxidation; DNL, *de novo* lipogenesis; ↑, proposed to increase; ↓, proposed to decrease; ?, unclear due to divergent findings.

Rodent studies consistently implicate ERα as the primary mediator of hepatic oestrogen signalling and a key regulator of IHTG accumulation. Female hepatic ERα knockout (KO) models consistently develop IHTG accumulation [135,139], while in female hepatic ERα KO mice subjected to OVX, E2 supplementation fails to protect against IHTG accumulation [125]. These observations demonstrate ERα may be a key mediator facilitating hepatic oestrogen signalling and IHTG dynamics in females. While limited, in male rodent models hepatic ERα KO mice have increased IHTG accumulation, which has been postulated to occur as a result of elevated gluconeogenesis and up-regulation of DNL genes (i.e., FAS and ACC) [140]. Furthermore, whole-body ERα KO mice of both sexes also develop insulin resistance, impaired glucose tolerance, and obesity [141], suggesting a systemic FA metabolic role for ERα [141]. ERα in rodents has been demonstrated to regulate hepatic FA metabolism in a sexually dimorphic manner when challenged with a high-fat diet (HFD). Following an HFD, female hepatic ERα KO mice accumulate IHTG to a greater extent compared with their littermate controls [142]. In contrast, both littermate controls and ERα KO male mice accumulate IHTG to a similar extent [142,143], indicating hepatic ERα does not protect males to the same extent as females against HFD-mediated IHTG accumulation. There are few studies focused on the metabolic roles of ERα in humans. ERα polymorphisms in both men and women are associated with increased type 2 diabetes prevalence and elevated fasting glucose [144–146]. A case report of a man lacking functional ERα demonstrated they had insulin resistance and dyslipidaemia [147], while hepatic ERα expression is reduced in men with compared with those without MASLD [148].

Collectively, these findings indicate that ERα is an important regulator of oestrogen-mediated hepatic FA metabolism, with impairments increasing IHTG accumulation risk in both sexes. However, in response to HFD, ERα serves as a greater protector against dietary FA-mediated IHTG accumulation in rodents; however, this has not been explored in humans.

Hepatic GPER1 knockout exacerbates IHTG accumulation in both male and female mice fed a HFD and high-fat high-cholesterol diet (HFHC), whereas GPER1 activation protects against the increasing IHTG accumulation, suggesting minimal sexual dimorphism in GPER-mediated metabolic signalling [149], even in response to substrate overload. Thus, GPER1 influences IHTG levels in rodents; however, this remains unclear in humans.

Further sex differences influencing IHTG accumulation exist through the aromatisation of testosterone to oestrogen. As E2 is produced in men by the aromatisation of testosterone, studies in humans have found that men with aromatase mutations in the liver display higher MASLD risk factors, including elevated plasma TG and reduced plasma high-density lipoprotein, while supplementation with oestrogen has been shown to reduce risk [150]. Moreover, postmenopausal women with breast cancer using aromatase inhibitors appear to have a higher risk of MASLD compared with postmenopausal women without MASLD, independent of BMI or diabetes status [151]. These findings are supported by rodent studies where aromatase gene knockout (AromKO) and castrated male mice develop hepatic steatosis, which can be rescued upon supplementation with E2 [148,152]; however, increased IHTG accumulation is not observed in female AromKO mice. The exact mechanisms by which E2 is protective in IHTG accumulation in males remain unclear. E2 supplementation in male AromKO mice improves hepatic mitochondrial function and permeability, suggesting enhanced hepatic FAO [153]. Taken together, these findings implicate aromatase as being involved in IHTG accumulation; however, this may play a larger role in males compared with females.

### Testosterone

Testosterone is the principal circulating androgen in both sexes. In men, it is synthesised from cholesterol in testicular Leydig cells, whereas in women it is produced primarily by the ovaries and adrenal glands. A key sex difference is production level, with circulating testosterone concentrations up to eight-fold higher in men than women [154]. Testosterone can be converted to dihydrotestosterone (DHT) via 5α-reductase or aromatised to oestradiol (E2). In circulation, only a small fraction exists as free testosterone, with the majority bound to sex hormone–binding globulin (SHBG) or albumin. Testosterone mediates its biological effects via androgen receptors (AR) in the liver; DHT binds to AR with higher affinity but cannot be aromatised. Unlike ERs, hepatic ARs are expressed in both sexes in humans and rodents, but in a sex- and age-dependent manner, with markedly higher hepatic expression reported in male compared with female rats that increases after puberty and declines with age [155,156].

Testosterone regulation of IHTG accumulation has been largely explored in male rodent models, while work in female rodent models remains sparse. Across multiple orchidectomy models, reduced testosterone consistently induces IHTG accumulation and hepatic steatosis, effects that are reversed by testosterone replacement, including under HFD conditions [157–162]. Similar phenotypes are observed in AR-deficient models, with some global AR knockout mice and liver-specific AR knockout (LARKO) mice displaying increased IHTG accumulation and hepatic steatosis compared with controls [163–165], although this is not universally found across global AR KO models [166,167]. Mechanistically in mice, testosterone deficiency or impaired AR signalling is associated with reduced gene expression of genes involved in VLDL-TG efflux (MTTP and ApoB100) and FAO (PPARα), and increases in DNL-associated genes (SREBP-1c and ACC); however, flux through these pathways remains unclear [165,168]. *In vitro*, testosterone treatment of male PHHs reduces intrahepatocellular TG by decreasing lipid droplet size and number [83], suggesting testosterone directly influences lipid droplet dynamics.

Human data support marked sex-specific effects (Table 2). In men, low circulating testosterone is associated with increased IHTG accumulation, visceral adiposity, MASLD prevalence and disease severity, while testosterone replacement in hypogonadal men improves liver-related parameters in some cohorts [169–172]. In contrast, in women, excess testosterone is associated with increased IHTG accumulation and VAT adiposity across pre- and postmenopausal states [173–175]. This is most evident in women with polycystic ovary syndrome (PCOS), where hyperandrogenism is linked to increased liver fat and MASLD risk [176,177]. Supporting a direct hepatic effect in women, testosterone increases lipid droplet size in female PHHs, contrasting its lipid-lowering effects in male PHHs [83]. Menopause, characterised by relative testosterone excess due to rapid oestrogen decline, may further contribute to increased IHTG accumulation in women [178,179].

Collectively, these findings indicate that testosterone protects against IHTG accumulation in men but promotes hepatic lipid accumulation in women, highlighting a strongly sex-dependent role for androgen signalling in hepatic FA metabolism and MASLD risk.

## Conclusion

**Figure 3:**
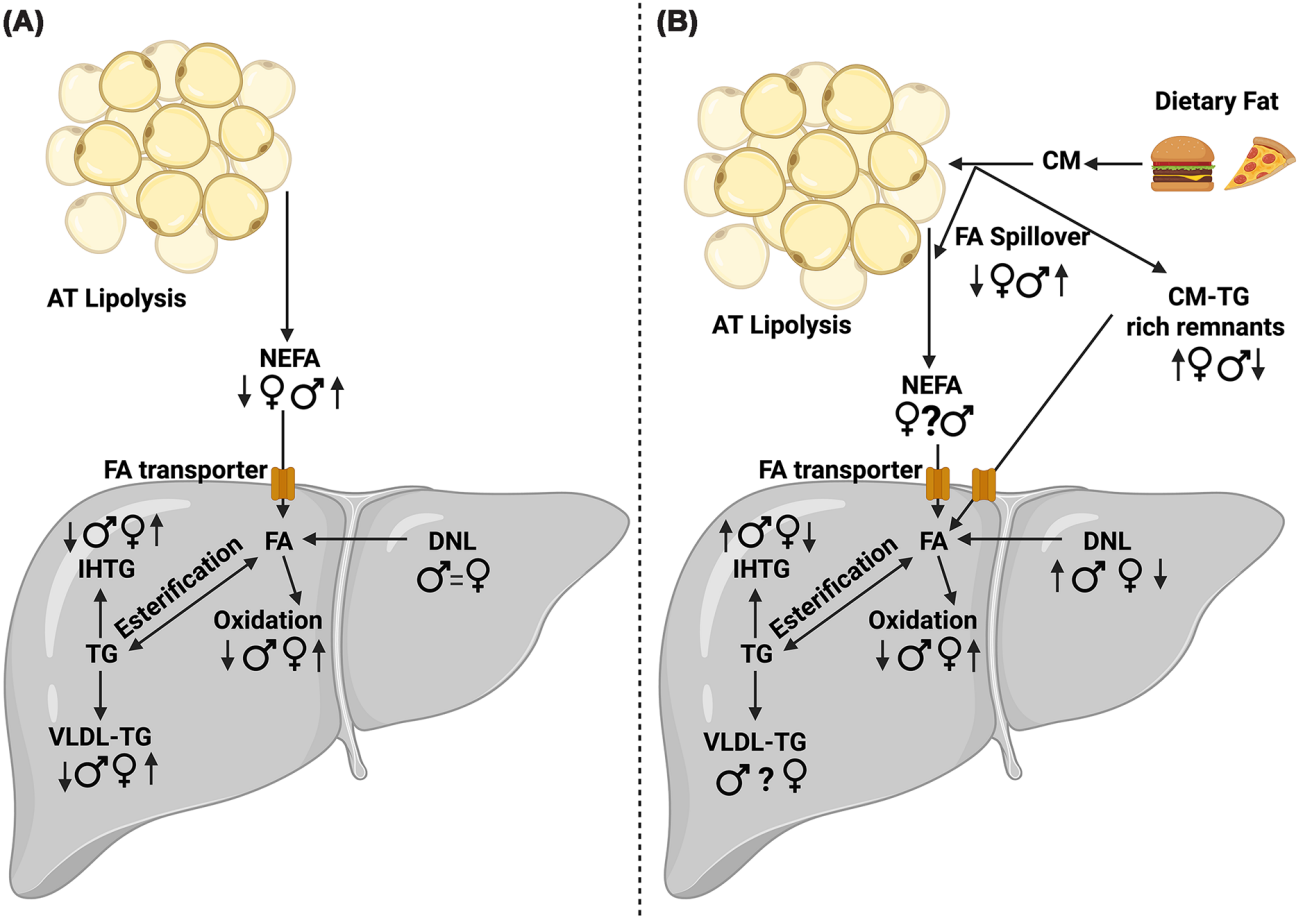
Summary of known sex differences in hepatic fat metabolism in the fasting and postprandial states. An overview of the proposed sex-specific differences in (**A**) fasting and (**B**) postprandial hepatic fatty acid metabolism in men and premenopausal women. Figure created in https://BioRender.com. Dennis, K. (2026) https://BioRender.com/6j0oah4. Symbols and abbreviations: ♀ premenopausal women; ♂ men; ?, unclear due to divergent findings; =, no difference between sexes; fatty acid, FA; DNL, *de novo* lipogenesis; VLDL, very‐low-density lipoprotein; IHTG, intrahepatic triglyceride accumulation.

From the available evidence, it appears that hepatic FA metabolism is sexually dimorphic in men and premenopausal women, with divergences in FA delivery to, intrahepatic FA synthesis and partitioning with, and disposal (through export of TG in VLDL and FAO) from the liver that contribute to sex-specific risk of IHTG accumulation and MASLD development (Figure 3). Changes in hormones with age, in particular the decline in oestrogens present post-menopause, appear to reduce sexual dimorphism in hepatic FA metabolism. Men generally exhibit hepatic metabolic phenotypes that favour FA partitioning towards esterification and storage, resulting in higher IHTG content. In contrast, premenopausal women appear to preferentially direct FAs towards oxidation and VLDL-TG efflux, thereby limiting IHTG accumulation; however, this hepatic metabolic profile is lost following the menopause in women. These sex-dependent divergences in hepatic FA metabolism are driven, at least in part, by sexually divergent patterns of hormonal regulation, including insulin, glucagon, and the sex hormones oestrogen and testosterone. Through their effects on systemic FA utilisation, adipose tissue distribution and metabolism, these hormonal differences collectively modulate FA flux to the liver, in addition to directly influencing intrahepatic FA metabolism in a sex-specific manner. Despite the recent advances in hepatic FA metabolism from *in vivo* human work and preclinical rodent models, the mechanisms governing sex-specific hepatic FA handling remain poorly defined in humans, particularly those underlying the metabolic shift observed during the transition into menopause in women. While not explored in humans, the presence of hepatic ERα may explain why premenopausal women have lower levels of IHTG accumulation and reduced MASLD risk compared with men. However, whether changes in ERα expression or function explain increased IHTG accumulation and MASLD risk in pre- compared with postmenopausal women remains unclear.

Given the potential role of oestrogen in women lowering IHTG accumulation and risk of MASLD, HRT has been considered as a preventative therapeutic option for postmenopausal women, although results are conflicting, with some showing improvement with HRT whereas others find minimal or no differences compared with control groups [118,180,181].

MASLD is increasingly recognised for consequences beyond the liver, including increased risk of type 2 diabetes and cardiovascular disease. Recent evidence indicates MASLD significantly increases the risk of heart failure, specifically heart failure with preserved ejection fraction [182], which is a well-characterised sexually dimorphic disease with higher incidence in older women [183]. Taken together, improved understanding of sexual dimorphism in hepatic FA metabolism is required for the development of sex-specific therapeutic strategies targeting dysregulated hepatic FA metabolism and MASLD, conferring benefits for cardiometabolic disease risk, which remains disproportionately understudied in women.

## Open Access

Open access for this article was enabled by the participation of the University of Oxford in an all-inclusive Read & Publish agreement with Portland Press and the Biochemical Society under an agreement with JISC.

## Abbreviations

ATGL: adipose triglyceride lipase
AR: androgen receptors
ApoB100: apolipoprotein B-100
AromKO: aromatase gene knockout
BMI: body mass index
DNL: *de novo* lipogenesis
DHT: dihydrotestosterone
FA: fatty acid
FAO: FA oxidation
GPER1: G-protein coupled oestrogen receptor 1
HL: hepatic lipase
HFD: high-fat diet
HFHC: high-fat high-cholesterol diet
IDLs: intermediate-density lipoproteins
IHTG: intrahepatic triglyceride
KO: knockout
LDL: low-density lipoprotein
MASLD: metabolic dysfunction-associated steatotic liver disease
OVX: ovariectomixed
PCOS: polycystic ovary syndrome
PET/CT: positron emission tomography/computed tomography
PHHs: primary human hepatocytes
SHBG: sex hormone binding globulin
SAT: subcutaneous adipose tissue
Sf: Svedberg flotation
TG: triglyceride
TRLs: TG-rich lipoproteins
VLDL: very-low-density lipoprotein
VAT: visceral adipose tissue
3-OHB: 3-hydroxybutyrate
